# A new perspective of the 2014/15 failed El Niño as seen from ocean salinity

**DOI:** 10.1038/s41598-019-38743-z

**Published:** 2019-02-25

**Authors:** J. Chi, Y. Du, Y. Zhang, X. Nie, P. Shi, T. Qu

**Affiliations:** 1 State Key Laboratory of Tropical Oceanography, South China Sea Institute of Oceanology, Chinese Academy of Sciences, Guangzhou, 510301 China; 2 University of Chinese Academy of Sciences, Beijing, 100049 China; 3 Joint Institute for Regional Earth System Science and Engineering, University of California, Los Angeles, CA 90095 USA; 4 Key Laboratory of Marine Science and Numerical Modeling, First Institute of Oceanography, Ministry of Natural Resources of the People’s Republic of China, Qingdao, 266000 China

## Abstract

This study investigates the 2014/15 failed El Niño using salinity from an ocean general circulation model. The results indicate that subsurface processes were especially strong in the summer of 2014 and they led to positive sea surface salinity anomalies in the central equatorial Pacific. The positive sea surface salinity anomalies induced a westward displacement of the sea surface salinity front that represents the eastern boundary of the western Pacific warm pool, preventing the warm surface water from shifting eastward as seen in a typical El Niño event. In the meantime, more salty water was transported equatorward by a strengthening subtropical cell in the South Pacific. The enhanced subsurface processes in the central equatorial Pacific conveyed the salinity anomalies of subtropical origin to the sea surface and were largely responsible for the sea surface salinity variability but had less impacts on sea surface temperature during the 2014/15 failed El Niño, suggesting some potential advantage of ocean salinity in the El Niño-Southern Oscillation prediction.

## Introduction

**Figure 1:**
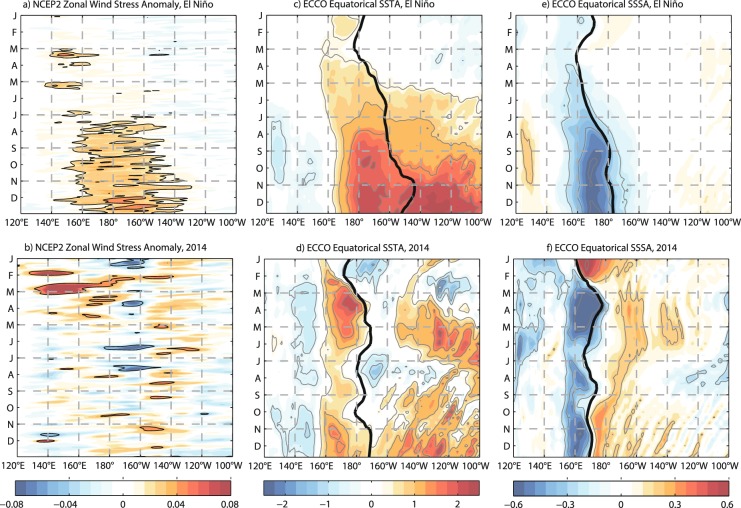
Anomalous wind stress, SST, and SSS along the equatorial Pacific from ECCO. (**a**,**b**) Zonal wind stress anomalies (N/m2). (**c**,**d**) SST anomalies (°C). (**e**, **f**) SSS anomalies (psu). (**a**,**c**,**e**) are a composite of El Niño events. (**b**,**d**,**f**) are 2014/15 failed El Niño events. The thick black lines denote the eastern edge of the warm pool (29 °C isotherm) in (**c**,**d**) and SSS front (34.8-psu isohaline) in (**e**,**f**).

The El Niño-Southern Oscillation (ENSO) is one of the most prominent phenomena in the world’s climate system^1–7^. In many cases, ENSO can be regarded as a highly damped oscillation triggered by atmospheric noises such as the westerly wind bursts in the western equatorial Pacific (Fig. 1a)^8–12^. In early 2014, as strong westerly wind bursts occurred in the western equatorial Pacific (Fig. 1b), an El Niño was highly anticipated by the community^13,14^. However, the El Niño condition quickly decayed as strong easterly winds developed in the following summer. From the point of view of ocean-atmosphere coupling, there is no doubt that the anomalous easterly winds play a critical role in preventing the warm surface water from shifting eastward as seen in a typical El Niño event^15–17^. However, since the easterly winds and zonal sea surface temperature (SST) gradients in the equatorial Pacific (Fig. 1) are tightly linked, it is difficult to say one is the causal mechanism of the other, and the so-called “egg-and-chicken” issue still remains.

This study provides a new look at the 2014/15 failed El Niño from ocean salinity. In the past decades, sea surface salinity (SSS) indices have been introduced to characterize ENSO^18–25^. During the developing phase of El Niño, SSS anomalies are remarkably negative in the western equatorial Pacific near the dateline, leading to an eastward movement of the SSS front (defined as the longitudinal location of the 34.8-psu isohaline) (Fig. 1e). The SSS front moves consistently with the eastern edge (defined as the longitudinal location of the 29 °C isotherm) of the western Pacific warm pool. The negative SSS anomalies in the western equatorial Pacific also result in a thicker barrier layer^26^, representing an enhanced surface stratification^24^. Investigating the processes that govern the SSS variability in the equatorial Pacific may help understand the ENSO evolution and prediction. Earlier studies have shown that, besides of horizontal advection and precipitation, subsurface processes also play an important role in modulating the SSS variability in the equatorial Pacific^23,27^. A good example of these subsurface processes is the resurfacing of South Pacific Tropical Water, which may contribute to the SSS variability at a rate equivalent to as much as 25% of the surface freshwater flux in the equatorial Pacific^28^.

Here we relate the SSS variability in the equatorial Pacific to changes in subtropical circulation during El Niño events, using results from an ocean state estimate of the consortium for Estimating the Circulation and Climate of the Ocean (ECCO)^29^. Examining the relationship between the SSS variability and equatorward transport of salinity through the Subtropical Cell (STC)^30^ allows us to reveal new characteristics of ENSO and to provide a novel explanation for the 2014/15 failed El Niño.

## Result

### Mixed Layer Salinity Budget

The mixed layer salinity (MLS) is a good proxy of SSS, and their correlation in the equatorial Pacific exceeds 0.99. We first compare the simulated MLS budget terms with observations to assess the ECCO’s performance in simulating the equatorial Pacific variability (Supplementary-Fig. 1). The MLS budget terms include the MLS tendency, surface forcing, horizontal advection, and subsurface processes (detailed in Method section). The contributions of surface forcing and horizontal advection from ECCO show similar patterns to those from observations, and this result is consistent with previous studies^27^^,^^31^. We also learn from the ECCO estimate that subsurface processes play an equally important role as surface forcing and horizontal advection in regulating the MLS tendency in the equatorial Pacific^27^.

**Figure 2:**
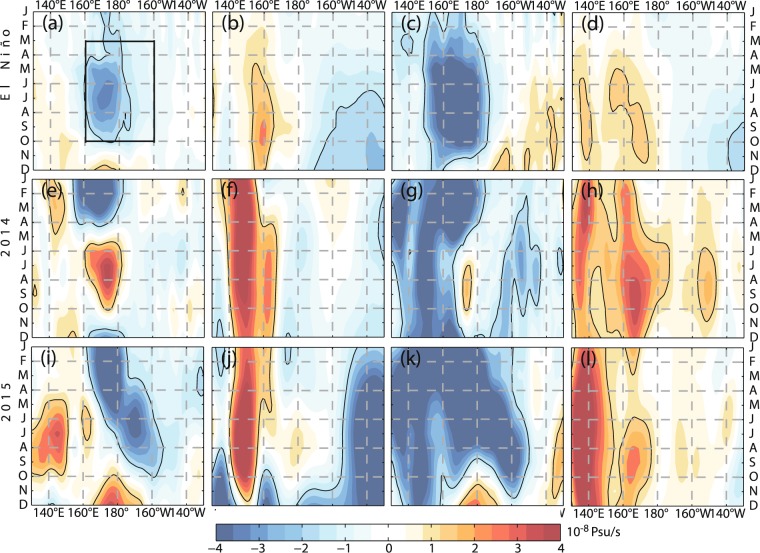
MLS budget terms along the equator from ECCO during (**a**–**d**) the composite, (**e**–**h**) the 2014/15 failed, and (**i**–**l**) 2015/16 El Niño events. There are four terms in the MLS budget: MLS tendency (**a**,**e**,**i**), surface forcing (**b**,**f**,**j**), horizontal advection (**c**,**g**,**k**), and subsurface processes (**d**,**h**,**l**). Seasonal cycles are removed. The black box in (**a**) ranges from 160°E-160°W and May-October.

**Figure 3:**
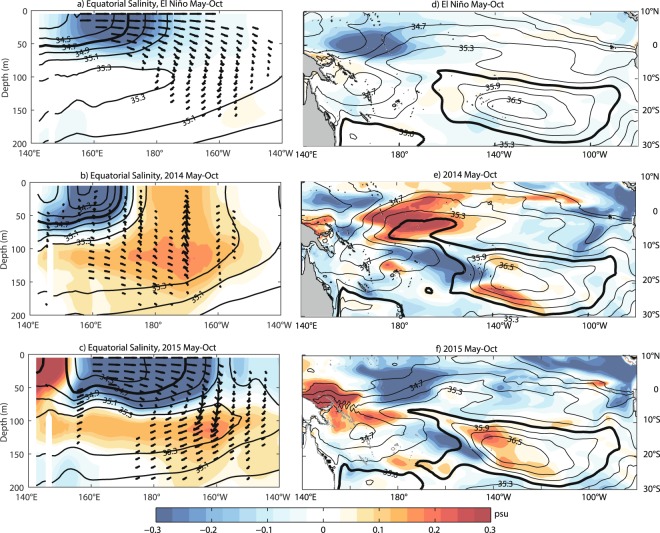
Vertical distribution of salinity (shading, contours) and velocity (vector) anomalies along the equator (3°S-3°N average, left column) and horizontal distribution of SSS (contours) and its anomalies (shading) during (**a**,**c**) the composite El Niño, (**b**,**d**) 2014 and (**c**,**f**) 2015. The black thick lines in (**a**–**c**) denote 34.7-psu isohaline, and the black thick lines in (**d**–**f**) denote the 35.6-psu isohaline.

To identify the processes responsible for the failure of the highly anticipated 2014/15 El Niño, we make a ensemble analysis (Supplementary-Fig. 2) and compare the MLS budget terms in 2014/15 with those from a composite (1994/95, 1997/98, 2002/03, 2004/05, 2006/07, 2009/10, and 2015/16) and an individual (2015/16) El Niño event (Fig. 2, Supplementary-Fig. 3). In the spring of 2014, as the SSS front moved eastward, the SSS anomaly was significantly negative near the dateline in the equatorial Pacific, which is also remarkable during the composite El Niño event (Fig. 1). The MLS tendency turned positive quickly after March and peaked (4.18 × 10^−8^ psu/s) in July 2014. In contrast, the negative anomalies (−1.47 × 10^−8^ psu/s, averaged between 160°E and 160°W from March to October) continue to occur during the composite El Niño event. The positive MLS tendency from March to October in 2014 was mainly caused by the subsurface processes (2.54 × 10^−8^ psu/s). During the composite El Niño event, the subsurface processes are less active (1.10 × 10^−8^ psu/s), and the enhanced horizontal advection is largely responsible for the negative MLS tendency. Though the subsurface processes (1.51 × 10^−8^ psu/s) were slightly stronger in 2015 (Fig. 2), they were overshadowed by the anomalously strong horizontal advection (−3.47 × 10^−8^ psu/s) in the equatorial Pacific.

Then, what subsurface processes controlled the SSS variability in 2014? These processes should be related to the vertical velocity and salinity gradient at the base of the mixed layer. For example, as a result of upwelling and upward directed vertical salinity gradient at 160°E-160°W (Fig. 3), a larger than normal amount of salty subsurface water entered the mixed layer through vertical entrainment, advection, and mixing during May to October 2014 (Supplementary-Fig. 3d). These vertical processes reduced the surface stratification and reversed the negative SSS tendencies in the central and western equatorial Pacific, and the SSS front was confined to the west of 170°E during most of 2014. With its eastern boundary characterized by the SSS front, the eastward displacement of the western Pacific warm pool was not visible. And as a consequence, the highly anticipated 2014/15 El Niño didn’t occur. The correspondence between the SSS front and SST anomalies in the equatorial Pacific may be related to surface stratification and the barrier layer^24,32^, but it is not the focus of the present work and will be investigated in a separate study. At least in a view of SSS indices, our result provides a new look at the 2014/15 failed El Niño and probably a new perspective for ENSO prediction.

### Connection with the Subtropical Cell

**Figure 4:**
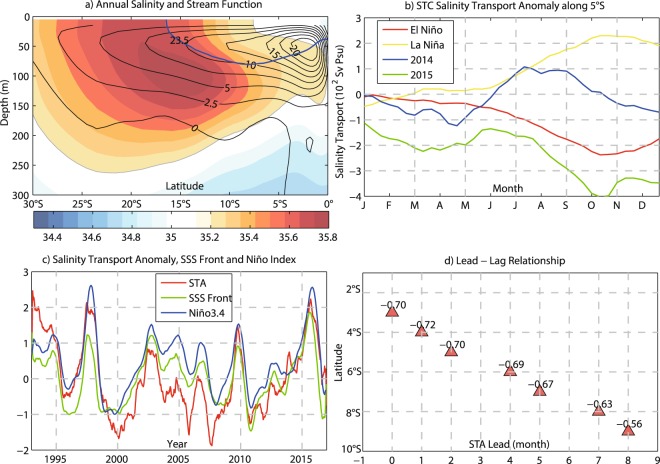
Salinity transport anomalies along subtropical cell and its relationship with Niño index. (**a**) Long-term mean salinity (shading, unit: psu) and stream function (contours, unit: Sv); (**b**) Equatorward STC salinity transport anomalies at 5°S; (**c**) Normalized time series of STC salinity transport anomalies at 5°S, SSS front, and Niño-3.4; (**d**) Lead-lag correlation of salinity transport anomalies at different latitudes with Niño-3.4. The number above each triangle denotes the correlation.

A recent study^28^ suggests that the resurfacing of subtropical water directly contributes to the SSS variability in the equatorial Pacific. We relate the resurfacing of subtropical water to the Subtropical Cell (STC)^30^. The STC is markedly evident in the long term mean meridional stream function (Fig. 4a). Simulated by ECCO, as much as 15 Sv (1 Sv = 10^6^ m^3^s^−1^) of subtropical water subducts to the upper thermocline between 10°S and 30°S of the South Pacific. A large portion of this water resurfaces in the equatorial Pacific^33^. The long-term mean equatorward salinity transport by the geostrophic flow at 5°S is 5.38 × 10^2^ Sv psu (15.21 Sv × 35.4 psu). During the composite El Niño event, the equatorward salinity transport simulated by ECCO decreases to 3.01 × 10^2^ Sv psu (Fig. 4b), less by as much as 44% than its long-term mean value.

The strengthening of the STC was tightly linked to the failure of the highly anticipated 2014/15 El Niño event. More subsurface water spread from the subtropical South Pacific into the central equatorial Pacific and upwelled during May-October 2014 (Fig. 3). The equatorward salinity transport at 5°S increased after April and peaked (6.48 × 10^2^ Sv psu) in July 2014, with an anomaly of more than 20% of its mean value (Fig. 4b). This positive salinity transport anomaly (STA) led the subsurface processes in the MLS budget by about 2 months. On interannual time scale, the equatorward STA at 5°S also leads the SSS front and Niño-3.4 index by about 2 months, with their correlation reaching −0.70 and −0.72, respectively (Fig. 4c). The equatorward STAs at lower latitudes are also highly correlated with Niño-3.4, but with shorter lead times (Fig. 4d). This lead-lag relationship between STA and Niño-3.4 confirms the connection of subtropical salinity anomalies and the equatorial SSS in the Pacific.

### Comparison with the Mixed Layer Heat Budget

Previous studies of ENSO have mostly focused on the thermal field. The SST signal of El Niño is most remarkable in the eastern equatorial Pacific, while SSS signal is strongest in the western and central equatorial Pacific (Fig. 1). On interannual time scale, salinity variability plays a significant role in modulating the surface density and mixed layer depth in the western and central equatorial Pacific^34^. To show the role of salinity variability in ENSO evolution, it is necessary to compare the mixed layer salinity budget with the mixed layer heat budget in the central equatorial Pacific.

The mixed layer heat budget terms during an El Niño event have their strongest signature in the eastern equatorial Pacific (Supplementary-Fig. 3). There, positive SST tendency is forced by positive horizontal advection, positive subsurface processes, and negative surface forcing. The year of 2014 seemed to be a developing period of the impending extreme 2015/16 El Niño event. However, the mixed layer salinity budget in the central equatorial Pacific gives a different perspective: an El Niño was anticipated in early 2014 but failed due to the anomalously strong subsurface processes during the rest of the year (Fig. 2). These subsurface processes play a more important role in generating the SSS variability than the SST variability in the central equatorial Pacific (Supplementary-Table 1).

**Figure 5:**
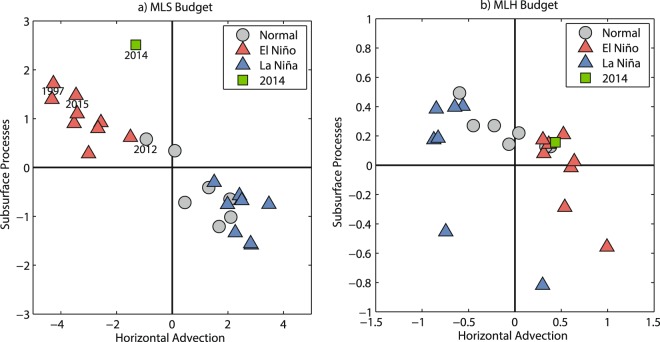
Scatter plot of horizontal advection and subsurface process anomalies during May-Oct over the central equatorial Pacific [3°S-3°N, 160°E-160°W]. (**a**) MLS budget. (**b**) MLH budget. The red (blue) triangles denote the El Niño (La Niña) development years. The green rectangles denote the budget terms in 2014. The gray circles denote the neutral status years. The units are 10^−8^ psu/s and 10^−7^ °C/s, respectively.

The El Niño events are clearly classified by the MLS budget analysis in Fig. 5a and Supplementary-Fig. 4. During a typical El Niño event, the anomalous eastward currents transport low-salinity water eastward from the fresh water pool and reduce the MLS in the central equatorial Pacific. Similarly, the contribution of horizontal advection to the MLS tendency in the central equatorial Pacific was also negative in 2014, but it was overshadowed by the subsurface processes. These subsurface processes brought relatively salty subsurface water to the surface and reduce the negative MLS tendency in the equatorial Pacific, preventing the SSS front or the eastern boundary of the western Pacific warm pool from moving further eastward. The effects of subsurface processes, however, cannot explain the 2014/15 failed El Niño by heat budget analysis alone (Fig. 5b). Above all, the mixed layer salinity budget analysis provides a unique opportunity to study ENSO.

## Discussions

Based on the MLS budget analysis from the ECCO estimate, this study suggests that the subsurface processes were unusually strong in the summer of 2014, which might contribute to the 2014/15 failed El Niño. The subsurface processes dominated the MLS tendency during May-November 2014 in the central equatorial Pacific. As more subsurface water from the subtropical South Pacific reached the equatorial Pacific through the STC, the subsurface processes were more efficient than horizontal advection in keeping a stable SSS front near the dateline. This stable SSS front prevented the warm and fresh surface water from shifting eastward, and was likely responsible for the failure of the highly anticipated 2014/15 El Niño. Furthermore, the good correspondence between the MLS tendency and the equatorward salinity transport by the STC confirms the subtropical influence on ENSO evolution. Interestingly, the impact of enhanced subsurface processes in the summer of 2014 was less obvious on SST, suggesting some potential advantage of ocean salinity in ENSO prediction. Above all, our study provides a new perspective of ENSO from ocean salinity, which we believe will be useful for ENSO prediction.

## Method

### Data

The description of the ECCO estimate refers to previous studies^35,36^. Only brief information is provided in the next two paragraphs. The model is based on the Massachusetts Institute of Technology general circulation model^37^, extending from 80°S to 80°N. The horizontal resolution is 1°, except within 20° of the equator, where its meridional grid spacing is gradually reduced to 0.3° within 10° of the equator. Vertical resolution varies from 10 m in the upper 150 m to 400 m near the bottom of the ocean. The model is spun up by time-mean seasonal wind stress along with air-sea flux of the Comprehensive Ocean-Atmosphere Data Set (COADS), then forced by those of the National Centers for Environmental Prediction (NCEP) reanalysis products^38^. To parameterize mesoscale eddy fluxes, the Redi isoneutral mixing scheme^39^ and the Gent and McWilliams parameterization^40^ are employed. The K-Profile Parameterization (KPP) vertical mixing scheme^41^ is employed for the mixing processes in the mixed layer.

We use results from the ECCO near real-time state estimate (ECCO-KFS dr080) distributed by Jet Propulsion Laboratory (JPL), which is assimilated by a partitioned Kalman filter and Rauch-Tung-Striebel (RTS) smoother^42^. To reduce state approximations in ECCO original output, sea level anomalies from satellites (TOPEX/Poseidon, Jason-1, and Jason-2) and temperature profiles from *in-situ* measurements (e.g., Argo, XBTs, and CTDs) are assimilated into the model. The filter assimilation improves the model state by large-scale adiabatic adjustments, but is not dynamically consistent. Therefore, the fixed-interval RTS smoother is applied to correct inaccuracies in the wind forcing. The model runs for the period from 1980 to present and relaxes SSS to climatological state with a 60-day relaxation coefficient. The Smoother version used for this study is dynamically consistent, and well suited for the salinity budget analyses. Previous studies suggested that the ocean’s general circulation and water properties simulated by ECCO are fairly consistent with observations^27,35,36,43–45^. The ECCO outputs for the period 1993–2016 used for this study are obtained from http://ecco.jpl.nasa.gov/external/.

Mixed Layer Salinity BudgetThe method is similar to recent studies^27,35^, and the following description is derived from these studies with modification. The time evolution of MLS in the equatorial Pacific is estimated by^46^

1 $$\frac{\partial [S]}{\partial t}=surface-{[{\nabla }_{H}\cdot (uS,vS)]}_{ml}+{[mixing]}_{H}+subsurface$$

2 $$subsurface=-\,\frac{1}{h}{\rm{\Delta }}S\frac{\partial h}{\partial t}-{[{\nabla }_{H}\cdot (uS,vS)]}_{induct}-[{\nabla }_{Z}(wS)]+{[mixing]}_{Z}$$

where the square bracket represents the depth average within the mixed layer. The mixed layer depth (MLD), *h*, is defined as the depth where the density increases from the surface value due to 0.2 °C temperature decrease. [*S*] and Δ*S* denote the MLS and the salinity jump across the base of the mixed layer; *u* and *v* are zonal and meridional components of velocity; the subscript *H* and *Z* denote horizontal and vertical components of a variable. The MLS tendency, $\frac{\partial [S]}{\partial t}$, attributes to three parts in this study: the surface forcing, horizontal advection, and subsurface processes. The surface forcing, the first term on the right hand side of eq. (1), consists of the effect of E - P and the surface relaxation in the model. The horizontal mixing is at least one order smaller than horizontal advection over the tropical Pacific in the mode result (not shown), so we combine these two terms and refer it as “horizontal advection” for simplicity of discussion^27^. The “subsurface” processes in eq. (2) consist of the entrainment (the first two terms), vertical advection (the third term), and subsurface component of mixing (the last term). Different from most earlier budget analysis, the vertical entrainment here can be either positive or negative, depending on the vertical salinity gradient as well as the horizontal MLD gradient. The [*mixing*]_*Z*_ consists of the vertical turbulent diffusion at the base of the mixed layer base, the GM mixing^40^ and the KPP nonlocal component within the mixed layer^41^. All terms of eqs (1) and (2) are computed at the model’s integration time step and archived as 30-day averages.

## Supplementary information

Supplementary Information
