# Supplementary material for: A new perspective of the 2014/15 failed El Niño as seen from ocean salinity
Source: Sci Rep. 2019 Feb 25;9:2720. doi: 10.1038/s41598-019-38743-z (PMC6389985; doi:10.1038/s41598-019-38743-z)
Supplement: Supplementary file 1 — Supplementary Information [file 41598_2019_38743_MOESM1_ESM.pdf]

Supplementary Information for

"A new perspective of the 2014/15 failed El Niño as seen from ocean salinity"

Jianwei CHI[1,2,3], Yan DU[1,2], Yuhong ZHANG[1], Xunwei NIE[4,3], Ping SHI[1] & Tangdong QU[3,4*]

[1]State Key Laboratory of Tropical Oceanography, South China Sea Institute of Oceanology, Chinese Academy of Sciences, Guangzhou 510301, China.

[2]University of Chinese Academy of Sciences, Beijing 100049, China.

[3]Joint Institute for Regional Earth System Science and Engineering, University of California, Los Angeles, CA, 90095, USA.

[4] Key Laboratory of Marine Science and Numerical Modeling, First Institute of Oceanography, Ministry of Natural Resources of the People's Republic of China, Qingdao 266000, China.

[*]Corresponding author: Tangdong Qu (tangdong@ucla.edu)

Supplementary-Fig.1

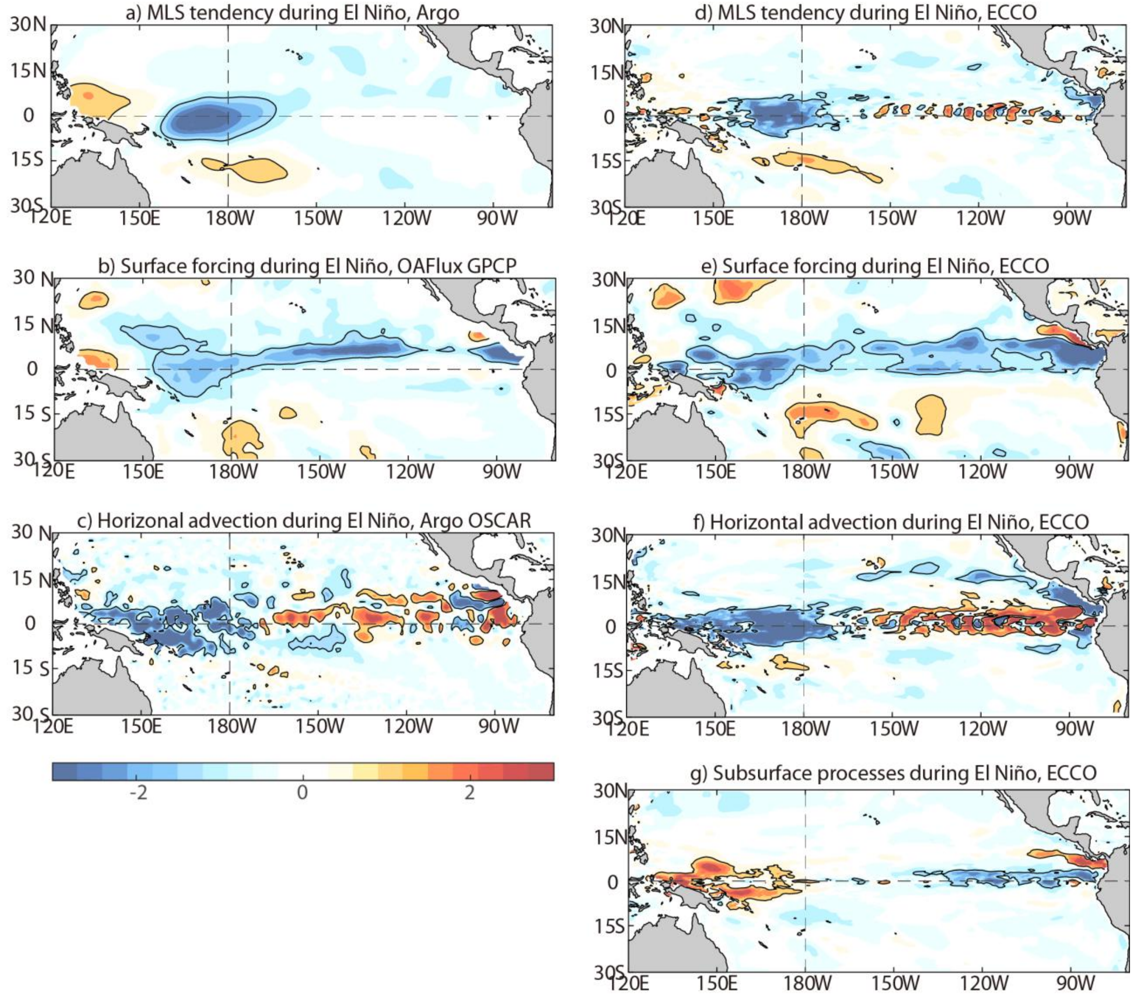

Supplementary-Fig.1. The mixed layer salinity budget terms during a composite El Niño (2006, 2009, 2015). The mean values (2005-2016) have been removed. (a, d) MLS tendency. (b, e) Surface forcing. (c, f) Horizontal advection. (g) Subsurface processes. The surface forcing from ECCO includes the effects of surface salinity flux (E-P) and surface relaxation. The sea surface horizontal velocity from OSCAR is used to estimate the horizontal advection in (c). The budget terms in (a), (b), and (c) are estimated with salinity from Argo, velocity from OSCAR, precipitation from GPCP2.3 and evaporation from OAFlux; (d), (e), (f), and (g) are estimated with ECCO.

Supplementary-Fig.2

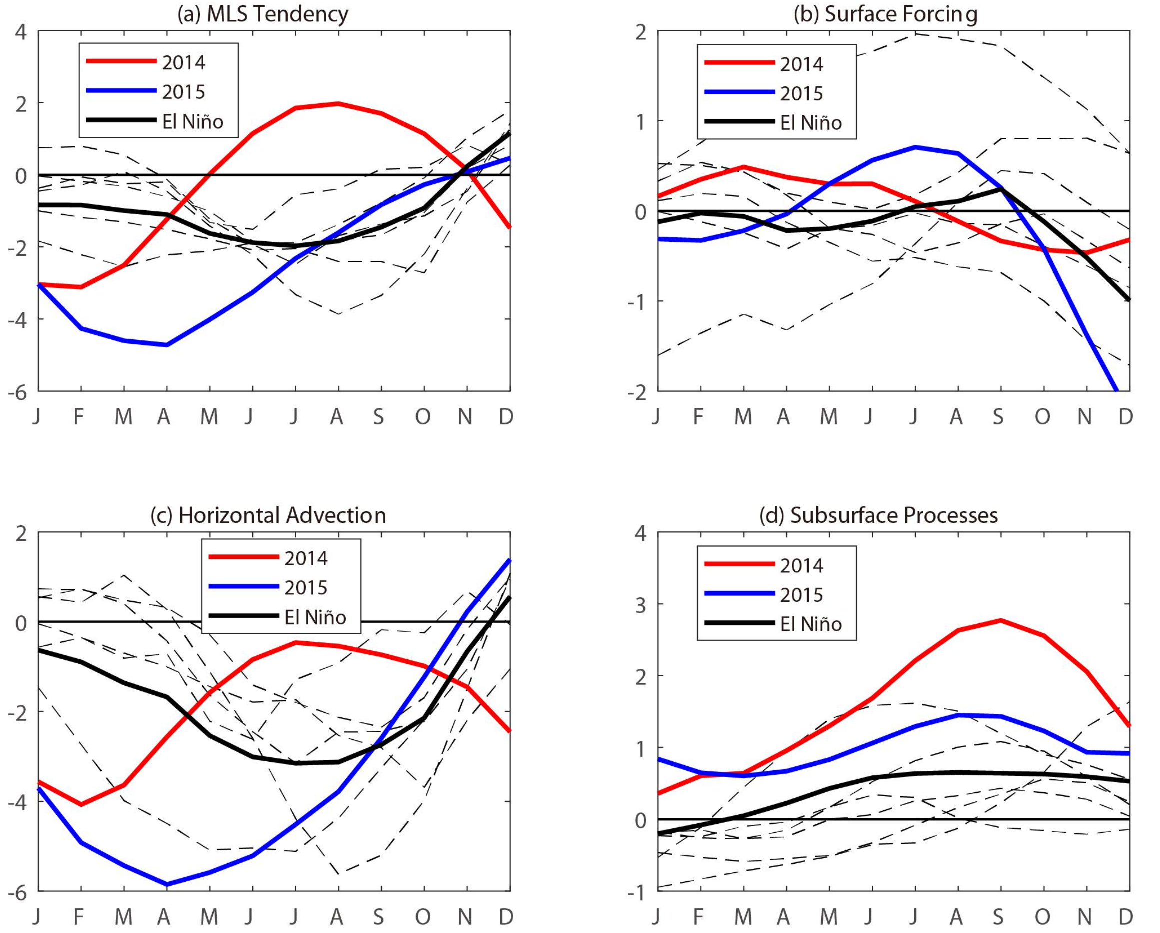

Supplementary-Fig.2 MLS budget terms in the central equatorial Pacific [3°S-3°N, 160°E-160°W]. The dashed black lines denote the budget terms during El Niño developing years (1994, 1997, 2002, 2004, 2006, and 2009); the red, blue, and black thick lines denote 2014, 2015, and the composite El Niño year, respectively. The units are 10^{-8}psu/s. Seasonal cycles are removed.

Supplementary-Fig.3

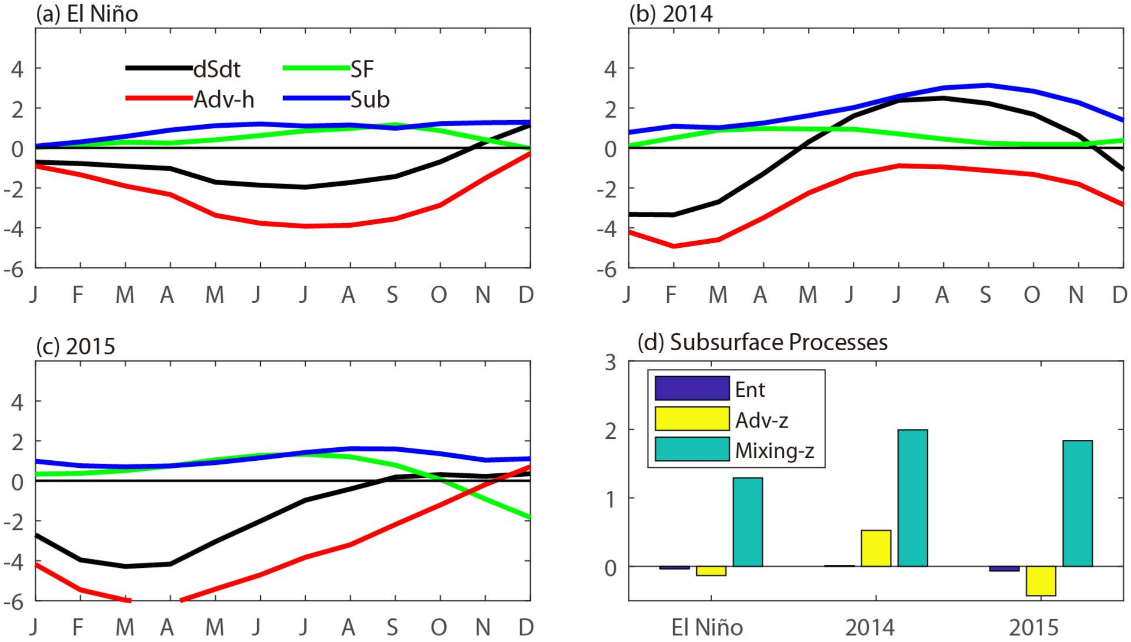

Supplementary-Fig.3 MLS budget terms in the central equatorial Pacific [3°S-3°N, 160°E-160°W] during El Niño (a), 2014 (b) and 2015 (c). The budget terms include the MLS tendency (dSdt), surface forcing (SF), horizontal advection (Adv-h), and subsurface processes (Sub). (d) The components of the subsurface processes averaged from May to October include the entrainment (Ent), vertical advection (Adv-z), and subsurface mixing (Mixing-z). The units are 10^{-8}psu/s. Seasonal cycles are removed.

Supplementary-Fig.4

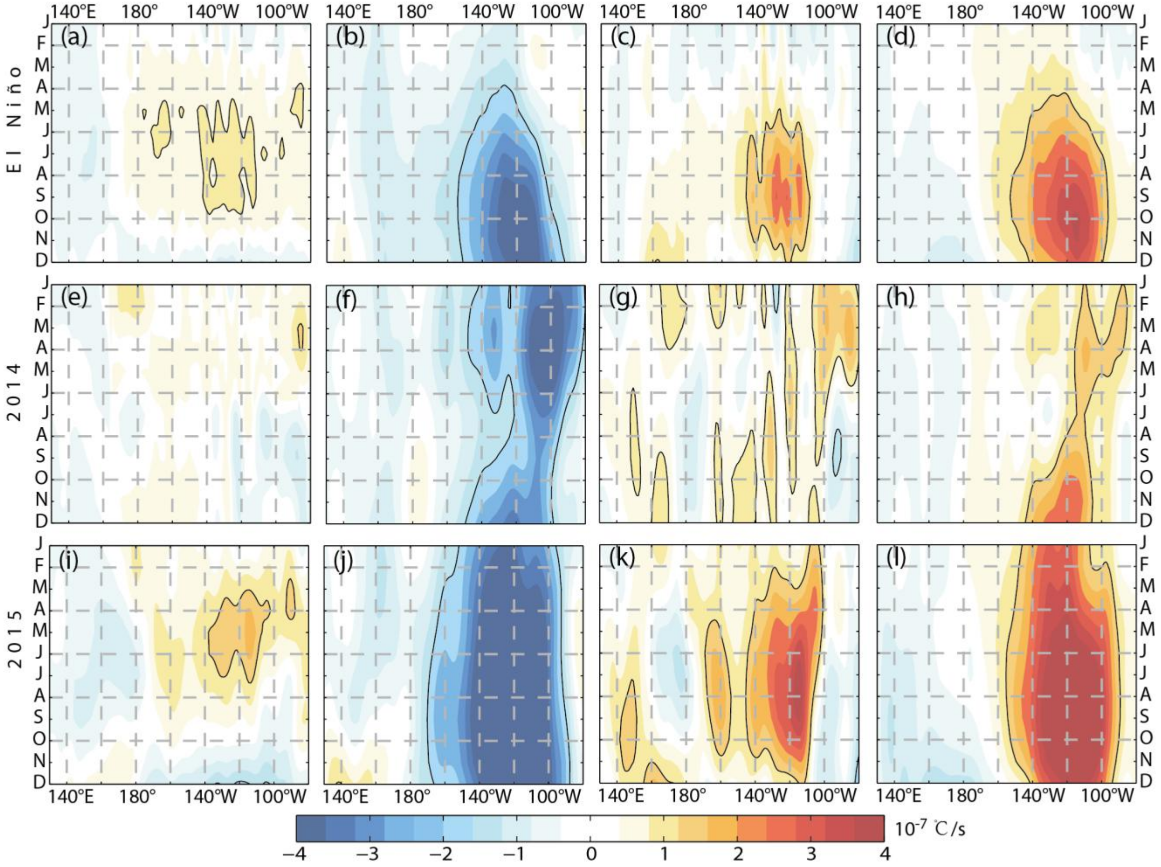

Supplementary-Fig.4 Mixed layer heat budget terms along the equator during a El Niño composite (a, b, c, d), 2014 (e, f, g, h), and 2015 (i, j, k, l). There are four terms in MLH budget: mixed layer heat tendency (a, e, i), surface forcing (b, f, j), horizontal advection (c, g, k) and subsurface processes (d, h, l). Seasonal cycles are removed.

51 **Supplementary-Fig.5**

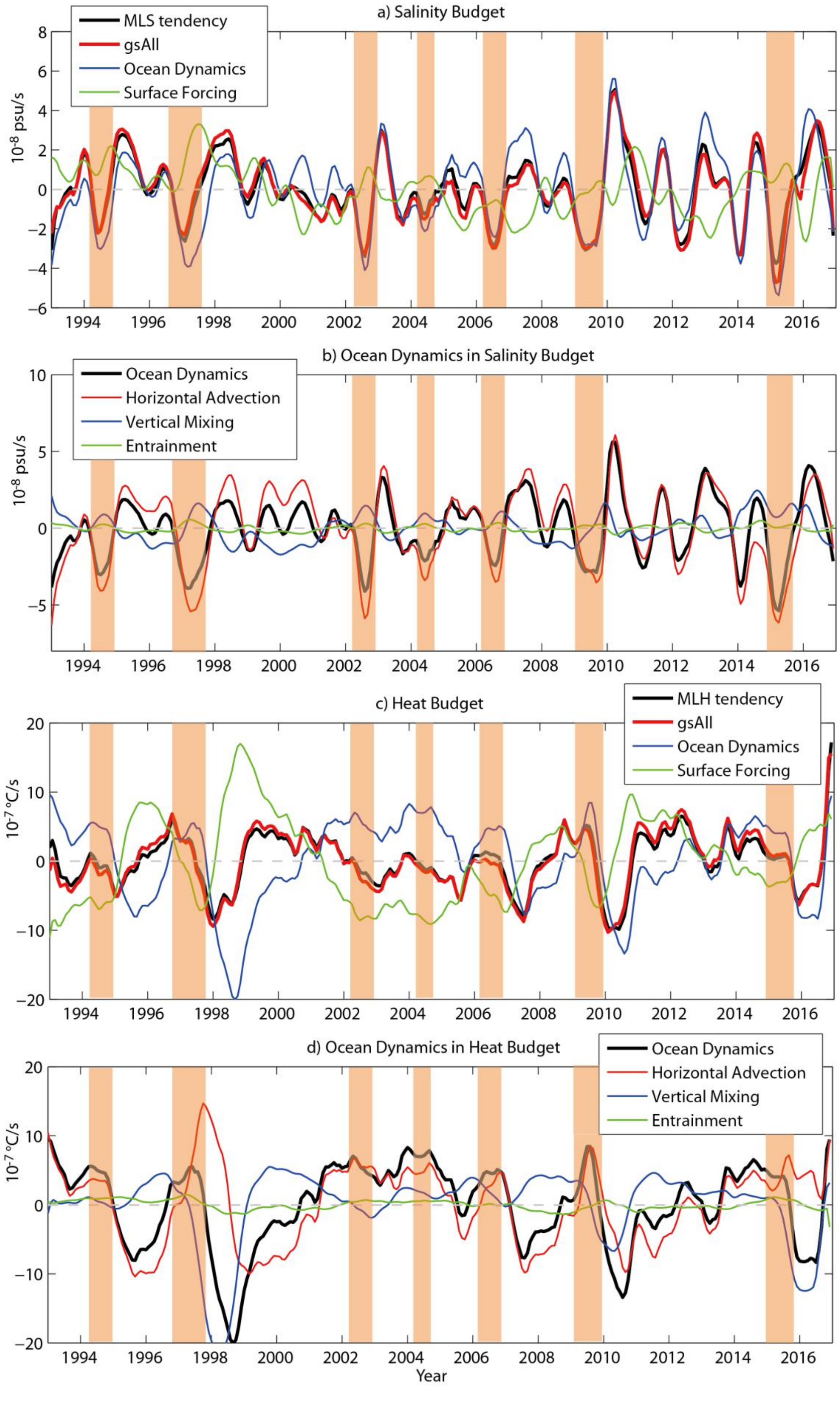

52

Supplementary-Fig.5 Interannual variations of a) the MLS and c) MLH budget terms and components due to ocean dynamics (b, d) in the central equatorial Pacific [3°S-3°N, 160°E-160°W]. A 13-month running mean filter has been applied to remove the mean seasonal cycle. Mean values have been removed before plotting the figures. The red bars denote the El Niño development periods.

Supplementary-Table 1

	MLS budget			MLH budget		
	El Niño	2014	2015	El Niño	2014	2015
Tendency	-1.47	1.72	-0.98	0.15	0.48	0.15
Sea Surface Forcing	0.83	0.49	0.95	-0.36	-0.13	-0.22
Horizonal Advection	-3.41	-1.32	-3.47	0.53	0.44	0.65
Subsurface Processes	1.10	2.54	1.51	-0.03	0.16	-0.29
Computational error	0.02	0.01	0.03	0.00	-0.01	-0.01

Supplementary-Table 1 MLS and MLH budget terms in the central equatorial Pacific. All values are averaged in [3°S-3°N, 160°E-160°W] during May-October. Seasonal cycles are removed. The units in the MLS and MLH budgets are 10^{-8} psu/s and 10^{-7} °C/s, respectively.
